# Illustration of Different Disease Transmission Routes in a Pig Trade Network by Monopartite and Bipartite Representation

**DOI:** 10.3390/ani10061071

**Published:** 2020-06-22

**Authors:** Kathrin Büttner, Joachim Krieter

**Affiliations:** Institute of Animal Breeding and Husbandry, Christian-Albrechts-University, Olshausenstraße 40, D-24098 Kiel, Germany; jkrieter@tierzucht.uni-kiel.de

**Keywords:** pig trade network, disease spread, transmission route, monopartite network, bipartite network, network analysis, epidemiological model

## Abstract

**Simple Summary:**

Besides direct animal movements between farms; indirect transmission routes of pathogens can have an immense impact on network structure and disease spread in animal trade networks. This study integrated these indirect transmission routes between farms via transport companies or feed supply as bipartite networks; which were compared to the monopartite animal movements network representing the direct transmission route. Both bipartite networks were projected on farm level to enable a comparison to the monopartite network. The number of edges increased immensely from the monopartite animal movements network to both projected networks. Thus, farms can be highly connected over indirect connections, although they are not directly trading animals. The ranking of the animals according to their centrality parameters, indicating their importance for the network, showed moderate correlations only between the animal movements and the transportation network. The epidemiological models based on the different network representations revealed significantly more infected farms for the networks including indirect transmission routes compared to the direct animal movements. Indirect transmission routes had an immense impact on the outcome of centrality parameters, as well as on the spreading process within the network. This knowledge is needed to understand disease spread and to establish reliable prevention and control measurements.

**Abstract:**

Besides the direct transport of animals, also indirect transmission routes, e.g., contact via contaminated vehicles, have to be considered. In this study, the transmission routes of a German pig trade network were illustrated as a monopartite animal movements network and two bipartite networks including information of the transport company and the feed producer which were projected on farm level (*n* = 866) to enable a comparison. The networks were investigated with the help of network analysis and formed the basis for epidemiological models to evaluate the impact of different transmission routes on network structure as well as on potential epidemic sizes. The number of edges increased immensely from the monopartite animal movements network to both projected networks. The median centrality parameters revealed clear differences between the three representations. Furthermore, moderate correlation coefficients ranging from 0.55 to 0.68 between the centrality values of the animal movements network and the projected transportation network were obtained. The epidemiological models revealed significantly more infected farms for both projected networks (70% to 100%) compared to the animal movements network (1%). The inclusion of indirect transmission routes had an immense impact on the outcome of centrality parameters as well as on the results of the epidemiological models.

## 1. Introduction

Network analysis has become a valuable framework for the analysis of disease transmission in animal trade networks [1,2,3,4,5,6]. Most of the studies focused thereby on the direct trade contacts between the agricultural premises as these contacts present a key transmission route especially for highly contagious diseases such as classical swine fever. Also, Fritzemeier et al. [7] stated that most of the secondary and follow-up outbreaks in Germany from 1993 to 1998 were caused by the trading of live animals. For the classical swine fever outbreak in The Netherlands from 1997 to 1998, Stegeman et al. [8] found comparable results. Likewise, Ribbens et al. [9] stated that the direct virus transmission via animal movements represents the largest contribution of the total disease transmission between farms before the first infected farm was detected. These direct animal movements are illustrated as monopartite networks including only one node type, e.g., the agricultural premises [10].

However, besides the direct trade contacts between the different farms, there are also other transmission routes that should be included in epidemiological studies in order to obtain a more realistic picture of disease transmission and to assess the importance of different transmission routes for the final epidemic size [11,12,13,14,15]. These transmission routes can be illustrated as bipartite networks, including two node sets, which are connected by edges entailing different kinds of meanings [10,16]. For instance, farms representing one node set can be connected over the transport company representing the other node set that actually ships the animals. Moreover, the actual truck, which is used for the transportation of the animals can be included in these bipartite networks. Furthermore, all other possible connections, which represent an indirect linkage between the farms, can be illustrated as bipartite networks, e.g., delivery of feed by different feed producers or person contact by e.g., the veterinarian. Especially, the vector truck is an important transmission route if the disinfection regulated by the Council Regulation (EC) No. 1/2005 was not carried out thoroughly. Thus, disease can be spread over different shipments and can, in this way, infect farms that do not directly trade with each other [17,18,19,20]. The same is true for the veterinarian or the feed supplier [7,11,21]. Over these vectors, more transmission routes can be analyzed instead of the pure direct animal movements between farms. 

Due to the fact that the inclusion of different transmission routes offers the more detailed illustration of the real probability of the disease spread, more recent studies also included indirect transmission routes into the network analysis and evaluated their impact on potential disease spread [5,11,13,18,19,21,22,23,24,25]. However, most of these studies focused on the inclusion of indirect disease transmission via livestock trucks. This can be explained by the fact that most of the attempts to account for indirect transmission routes are hindered due to low data availability [21]. Since the introduction of the EU directive 2000/15/EC, all animal movements have to be recorded in order to be able to perform backward tracing and to identify the primary outbreak in the case of an epidemic. However, this only includes the direct trade contacts between farms and does not include information about other transmission routes, e.g., truck rounds, veterinarian or feed supply, person contact, and shared equipment, which also complicate the analysis of indirect transmission routes within the animal trade networks. 

Another reason for the lower number of studies dealing with indirect transmission routes in animal trade networks can be that the analysis of monopartite networks is much more straight forward compared to the analysis of bipartite networks. Furthermore, for bipartite networks, the tools for analysis are still under development so that they do not offer the huge toolbox already existing for monopartite network analysis [10,16].

Although there are still problems regarding the data availability, as well as the methodological framework, for the best possible realistic overview, all important transmission routes should be considered in the investigations and their impact on the outcome of network analysis as well as on the potential epidemic size have to be evaluated. Thus, the aim of the present study was to include additional transmission routes as bipartite networks and to evaluate the impact on network parameters. Besides the effects of different transmission routes on the outcome of network analysis, their impact on the results of the spreading processes within the network should be evaluated. In this study, we focused not on a specific disease, but mainly on the effect of the network structure on the spreading processes within the network. For this study, besides the direct trade contacts, the bipartite networks considering the transportation as well as feed supply information were analyzed and compared to ascertain which network representation provides the best information. To the authors’ knowledge, this is the first study for the pig trade network in Germany, which investigates also indirect transmission routes with the help of network analysis. Furthermore, the impact of indirect connections between farms via feed producers was not yet evaluated. Thus, the present study provides deeper insights into the transmission routes within the pig trade network, which are needed in order to better understand potential disease spread and to establish appropriate disease control and prevention measures.

## 2. Materials and Methods 

### 2.1. Data

This study analyzed contact data from a producer community in Northern Germany. The observation period encompassed the years 2013 and 2014. The data included the date of the movement, the supplier, the purchaser, as well as the number and type of delivered livestock. Furthermore, additional information about the transport company for each shipment of animals as well as the feed supply, e.g., information about the feed producer of the farms, was available. Data on feed supply were recorded for a transparency program of a large food retailer to provide more information about the meat products to the consumers (e.g., producer, animal husbandry, feed, transportation, and geographical location of the farm), indicating that information about the feed supply was not accessible for all farms. Only the information about the transport company and the feed producer was available and not the vehicle that transported the animals or the feed. Moreover, the data included information on the type of each farm. 

### 2.2. Network Analysis

The transmission routes can be represented by nodes and edges forming a so-called network [10,16]. For instance, in animal trade networks focusing on the direct animal movements between the farms, the farms are the nodes, which are connected by edges if two farms are trading animals with each other. In this example, the edges have a clear direction pointing from the supplier to the purchaser of the animals. Thus, directed edges can only be passed in this given direction. This kind of network and the edges included in it are called directed. If no clear orientation of the edges is given, the network and its edges are called undirected.

#### 2.2.1. Monopartite vs. Bipartite Network Representation

Besides the above-given description of a network and its elements (nodes and edges), two types of network representations have to be differentiated: monopartite and bipartite networks.

A monopartite network includes only one set of nodes [10,16]. In the case of the pork supply chain, one monopartite representation can be the direct connection between two farms when they are trading animals with each other. Here, only one node set, namely the farms, is present in the network. 

In contrast to monopartite networks, in a bipartite network, there are two node sets. Only between nodes belonging to different node sets there can be a connection [10,16]. This means, besides the direct animal movements, also indirect connections between the farms, e.g., via livestock trucks, veterinarians, or feed supply, can be illustrated. Here, the farms represent one node set and the other node set might be e.g., the transport company, which organized the transport of the animals between two farms. Thus, the farms are not directly connected anymore, but indirectly over the second node set, e.g., the transport company.

#### 2.2.2. Network Construction

**Figure 1:**
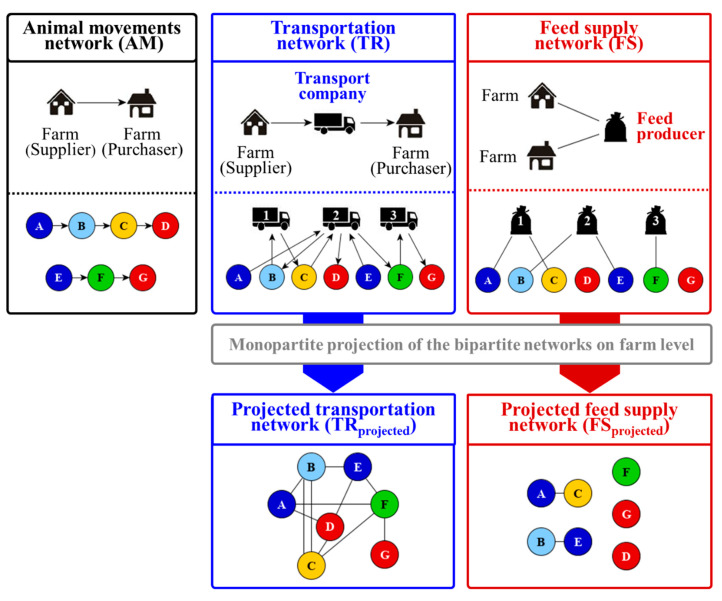
Network representations used in the present study. Monopartite network of animal movements between farms (black), bipartite transportation network (blue) and feed supply network (red). For both bipartite network representations, the monopartite projection on the farm level is illustrated.

Data on direct animal movements, transport companies, as well as feed supply, were analyzed in the present study. In this paragraph, the construction of different network representations based on the available data is illustrated and explained in order to evaluate possible transmission routes in the pig trade network. Figure 1 provides a detailed description of the different network representations with small examples characterizing the features and the construction of each network representation.

In the first instance, three network representations were constructed out of the available data basis: the monopartite animal movements network (AM), the bipartite transportation network (TR) (including only information about the farms and the transport companies) and the bipartite feed supply network (FS) (including only information about the connections between farms and feed producers). The monopartite animal movements network includes the direct trading activities between the farms, which implies that there is only one node set present in the network. In contrast to this, both bipartite network representations allow the inclusion of also indirect transmission routes into the network analysis including two node sets. For the transportation network, the farms are indirectly connected with each other over the transport company organizing the transport of the animals from the supplier to the purchaser. In the feed supply network, the farms are indirectly connected with each other over the feed producer delivering feed to each farm.

In order to be able to compare the direct trade contacts with the indirect transmission routes, which means to compare the connections between the farms in the monopartite network with the bipartite networks, both bipartite network representations were projected on farm level. These projections illustrate the indirect connections which are possible over the transport company or the feed producer. The small example networks provided in Figure 1 show the process of projection for both bipartite networks.

To create the monopartite projection on farm level for the bipartite transportation network (TR_projected_), a connection between the farms is drawn, if they are connected to the same transport company, e.g., farm A is connected to farm F because transport company two is used for the animal transport between these two farms with farm A as the supplier of the animals and farm F as purchaser (Figure 1). This specific connection is not portrayed in the monopartite animal movements network, thus, focus on the direct trade contacts might miss possible indirect transmission routes. For the projection of the bipartite transportation network, the direction of the edges of the bipartite network were considered. In order to allow re-entry of pathogens into the pork supply chain via contaminated livestock trucks, edge directions were removed after the projection for the projected transportation network. 

Similarly, the projection of the bipartite feed supply network was carried out. Because no information about the routes of the single feed deliveries was known, the bipartite feed supply network was considered undirected. Thus, also its projection on farm level (FS_projected_) was undirected. As can be seen in the small example network in Figure 1, not all farms were connected and thus isolated in the projection because they do not share the same feed producer. This was also the case for the projected feed supply network analyzed in the present study. Here, about 85% of the farms were isolated. Due to the high number of isolated farms, all calculations concerning the general network and centrality parameters were performed for the projected feed supply network with and without isolated farms.

After the projection, both projected network representations can be analyzed with the whole methodological framework existing for monopartite networks. Furthermore, the projection allows a comparison of the indirect transmission routes with the direct trade contacts between the farms.

#### 2.2.3. Static Aggregation

All different network representations illustrate static aggregated networks. A static aggregated network was constructed out of all daily records. This means for the animal movement network that repeated trade contacts between the same farms during the observation period were aggregated to a single one. For the other network types, the same procedure was carried out accordingly.

#### 2.2.4. General Network Parameters and Centrality Parameters

Network analysis provides a huge toolbox for the analysis of the whole group structure on a network level, as well as of the position of each node in the network on a node level. Network level parameters are called general network parameters and node level parameters are centrality parameters. All parameters were calculated using the Python module NetworkX [26]. Table 1 gives a short description of the general network and centrality parameters used in the present study.

### 2.3. Hypothetical Simulation of the Spreading Processes within a Pig Trade Network

In the present study, disease transmission was modeled using a simple SIR (susceptible–infected–recovered) model which was implemented in Python 3.4.2 [31]. No specific disease was simulated but the effect of the different network structures on the epidemic size. Thus, the present simulation model represents a hypothetical approach to evaluate the impact of the network structure and the included transmission route on disease spread. Each farm represented a single epidemiological unit, which was connected according to the three different network representations: animal movement network, projected transportation network, and projected feed supply network. In order to allow a further disease spread for the projected transportation network over the transportation companies, the edge directions were removed for the simulation.

Independent of the chosen network representation, each farm could be in three basic states: susceptible, infected or recovered (removed). Removed does not necessarily mean depopulation of the whole farm, but can also mean trade restrictions of the farms or immunity. In all cases, the farms are removed from the trade network and thus cannot spread the disease to other farms. At the beginning of the simulation, all farms were in the susceptible state. The epidemic started with a single infected farm chosen uniformly at random from the whole number of farms in the network. The successors for the directed animal movements network and the direct neighbors for the projected transportation network and the feed supply network became themselves infected by different transmission probabilities (T = 0.1, 0.2, ..., 0.9, 0.95). For each of the newly infected farms, their successors or neighbors, respectively, were determined and became again infected within the above-described procedure. A farm immediately became recovered, respectively, removed, after its successors or neighbors, respectively, had been determined. Removed farms played no further part in the course of the epidemic. No uncertainty or variability in the detection was implemented. Thus, the detection of an infected farm was assumed instantaneous. The epidemic ended when there were no more successors remaining in the network with the state susceptible. Furthermore, also no temporal aspects were included in the model. Due to the fact that only the farms were considered as epidemiological models no further statements about the removal of transport companies or feed producers can be made.

For each network representation and transmission probability the simulation model was run for 10,000 iterations, resulting in 300,000 iterations in total (3 network representations (AM—animal movements network, TR_projected_—projected transportation network, FS_projected_—projected feed supply network), 10 transmission probabilities (T = 0.1, 0.2, ..., 0.9, 0.95)). For each iteration, the number of susceptible, infected, and recovered farms were recorded.

Generalized linear models were calculated using the GLIMMIX procedure in SAS (Statistical Analysis System) in order to evaluate the effect of the network representation (AM, TR_projected_, and FS_projected_) and the transmission probability (T = 0.1, 0.2, …, 0.95) on the number of infected farms. Thus, the effect of the kind of contact (e.g., direct or indirect), as well as the probability for infection was tested on the size of the epidemic (e.g., number of infected farms) [32]. Only iterations resulting in more than one infected farm were kept in the data set for further analyses. In general, the fixed effects named above were added stepwise to the models, as well as the interaction between the two fixed effects were tested. The Akaike’s information criteria corrected (AICC) [33] and the Bayesian information criteria (BIC) [34] were used to compare the different models. The model with the smallest AICC and BIC values was chosen. The best model fit was obtained with an exponential distribution and a log-link function. All fixed effects (network representation and transmission probability), as well as the interaction between network representation and transmission probability remained in the final model. Significant differences in the least square means (LSMeans) were calculated and adjusted by the Bonferroni–Holm correction [35].

## 3. Results

### 3.1. Characterization of the Different Network Representations

#### 3.1.1. Monopartite Animal Movements Network (AM)

**Figure 2:**
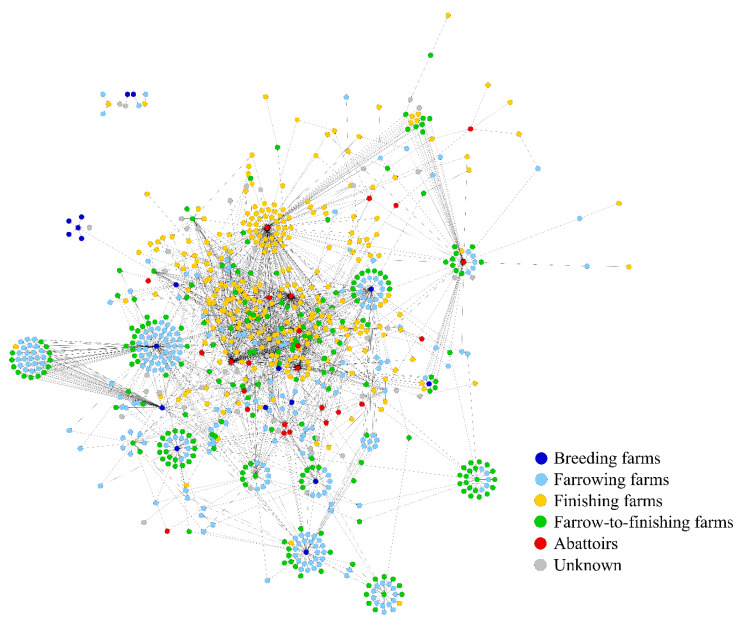
Monopartite animal movements network (AM) of a pork supply chain in Northern Germany covering the observation period from 2013 to 2014.

The monopartite animal movements network analyzed in the present study consists of a total of 866 farms, which are connected by 1884 static aggregated directed trade contacts between the farms, which means that repeated trade contacts between the same farms during the observation period were aggregated to a single one. The 866 farms can be categorized into 18 breeding farms, 265 farrowing farms, 281 finishing farms, 229 farrow-to-finishing farms, 25 abattoirs, and 48 farms with unknown farm type (due to missing information). Figure 2 illustrates the monopartite and directed animal movements network.

#### 3.1.2. Bipartite Transportation Network (TR) and Its Projection on Farm Level (TR_projected_)

The bipartite transportation network (TR) analyzed in the present study consists of a total of 1250 nodes that can be separated into 866 farms, which are identical with the animal movements network and 384 nodes which are responsible for the transportation of the animals. These 384 nodes can be categorized by 21 actual transport companies and 363 farms, which are also transporting animals. In total, there are 2973 edges of which 64% were performed by the actual transport companies. A Wilcoxon signed rank test indicated that the median number of shipments per transport company with 78 (range: 2–430) was significantly higher compared to the median number of shipments per farm which organized the transport by their own with 2 (range: 2–18). In this network, there are only edges between nodes of different sets, implying that the supplier is not directly connected to its purchaser but connected to the node that manages the transport of the animals (Figure 1). 

**Figure 3:**
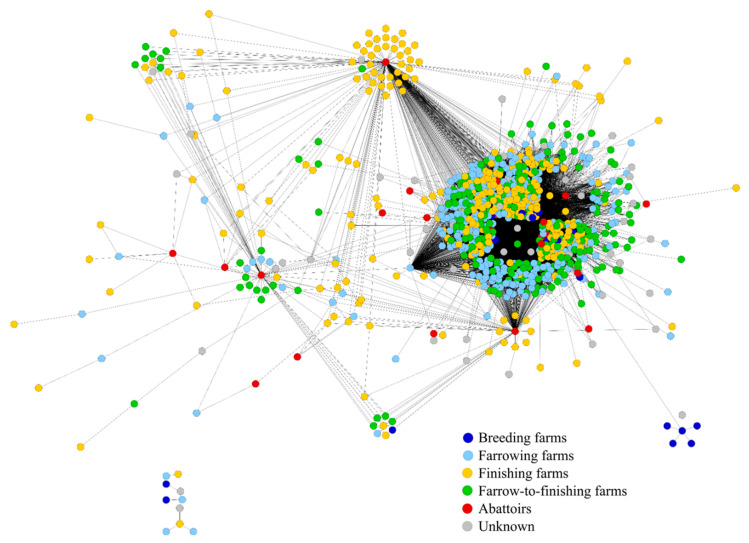
Projected transportation network (TR_projected_) of a producer community in Northern Germany covering the observation period from 2013 to 2014.

The projected transportation network (TR_projected_) consists of the same 866 farms similar to the animal movements network that were connected by 29,062 undirected edges. Figure 3 gives an illustration of the projected transportation network.

#### 3.1.3. Bipartite Feed Supply Network (FS) and Its Projection on Farm Level (FS_projected_).

The bipartite feed supply network analyzed in the present study consists of a total of 873 nodes. Here, the same 866 farms of the monopartite animal movements network constitute the first node set and seven feed producers constitute the second node set. Due to the fact that for this network representation no clear direction of the edges can be determined, the network is considered undirected with 178 edges connecting farms and their feed producers. One specification of this type of network is that for only 130 farms information about their feed producers was available, which resulted in 736 isolated farms. The non-isolated farms are of the following farm types: 8 farrowing farms, 83 finishing farms, and 39 farrow-to-finishing farms. 

**Figure 4:**
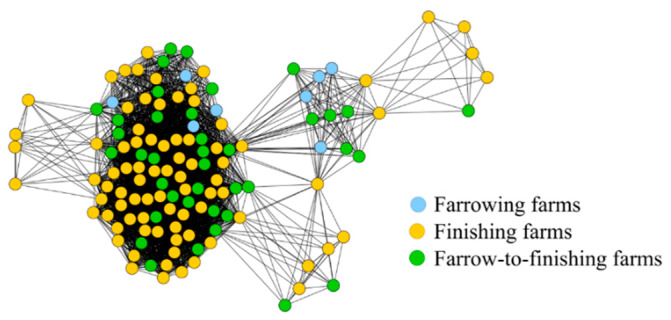
Projected feed supply network (FS_projected_) of a pork supply chain in Northern Germany covering the observation period from 2013 to 2014. Isolated nodes are not illustrated.

The projected feed supply network has the same configuration of number of farms, farm types, and isolated farms compared to the bipartite feed supply network. The 866 farms were connected by 3284 edges. Figure 4 illustrates the projected feed supply network. The figure was drawn without isolated nodes to enhance the clarity of the graphical illustration.

### 3.2. General Network Parameters and Centrality Parameters

In the following, the two projected network representations (TR_projected_ and FS_projected_) were compared to the monopartite animal movement network (AM) in order to evaluate the effect of different transmission routes on the general network and centrality parameters. Due to the high number of isolated farms in the projected feed supply network, the calculations were performed with and without isolates for this network representation.

#### 3.2.1. General Network Parameters

Table 2 illustrates the general network parameters for the monopartite animal movements network (AM) and the two projected network representations (TR_projected_ and FS_projected_). All three network representations were very sparsely connected with densities ranging from 0.003 to 0.075. Only the projected feed supply network had a higher density of 0.39 when the isolated farms were not considered for the calculation of the general network parameters. Although the networks were sparsely connected, the number of network components was very low, with only five network components for the animal movements and the projected transportation network and one for the projected feed supply network neglecting the isolated nodes. Furthermore, for these network representations, almost all nodes were part of the largest network component. These results can further be confirmed by the low values for the fragmentation. Only the projected feed supply network has a fragmentation close to one if the isolated nodes are included in the calculation of the general network parameters. The animal movements network had the largest number of detected communities with 18, followed by the projected transportation network with eleven communities, and in the projected feed supply network without isolated nodes five communities could be detected. Especially for the feed supply network, the five communities can clearly be identified in Figure 4. The maximum modularity Q_max_ varied between 0.24 and 0.52.

#### 3.2.2. Centrality Parameters

Table 3 illustrates the median centrality parameters for the three network representations animal movement network (AM), projected transportation network (TR_projected_), and projected feed supply network (FS_projected_). Comparable with the general network parameters, for the projected feed supply network, the centrality parameters were calculated with and without isolates. 

A Kruskal–Wallis test indicated that all three network representations had a significantly different median degree and median closeness values (*p* ≤ 0.05). With the lowest values for the animal movements network, followed by the projected transportation network and the highest values for the projected feed supply network without isolated nodes. For the betweenness, although the values were very low for all three network representations, the highest median betweenness values were obtained for the projected transportation network which were significantly different from the animal movements network and the projected feed supply network without isolated nodes.

### 3.3. Spearman Rank Correlation between the Centrality Parameters of the Different Network Representations

Table 4 illustrates the Spearman rank correlation coefficients between the centrality parameters of the animal movements network and both projected network representations for the undirected centrality parameters (degree, betweenness, and closeness). The projected transportation network demonstrated for all centrality parameters moderate correlation coefficients ranging from 0.55 to 0.68. For the projected feed supply network, the correlation coefficients were moderate for degree and closeness ranging from 0.41 to 0.45 and low for betweenness. If the isolated farms were removed from the network prior to the calculation of the correlation coefficients, no significant correlations with the animal movements network were detected.

### 3.4. Hypothetical Simulation of the Spreading Processes within a Pig Trade Network

**Figure 5:**
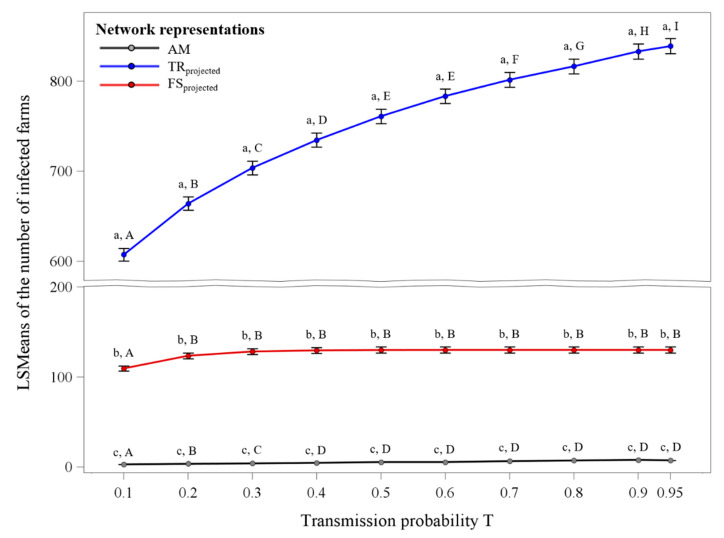
LSMeans ± standard error of the number of infected farms in dependence of the three different network representations and the transmission probability. (a–c) Significant differences between the network representations within each transmission probability. (A–I) Significant differences between the transmission probabilities within each network representation (*p* ≤ 0.05).

The results of the statistical model showed that all fixed effects, as well as the interaction between network representation and transmission probability, were significant (*p* ≤ 0.05). Figure 5 illustrates the LSMeans ± standard error of the number of infected farms for the interaction between network representation and transmission probability. It could clearly be seen that within each transmission probability, the differences between each network representation were significant (*p* ≤ 0.05). The projected transportation network revealed the highest number of infected farms ranging from 607 to 839, followed by the projected feed supply network with LSMeans ranging from 109 to 130 infected farms. The lowest LSMeans were detected for the animal movements network with values ranging from three to eight infected farms. 

Furthermore, the projected transportation network showed an increasing number of infected farms with increasing transmission probability, whereas for the other two network representations this holds only true for low transmission probabilities until T = 0.3.

## 4. Discussion

The aim of the present study was to evaluate the impact of the inclusion of additional transmission routes on the outcome of network analysis and epidemiological models. Indirect transmission routes between the farms’ information about transportation and feed supply, respectively, was included in the analysis as bipartite networks. These bipartite networks were then projected to monopartite networks on farm level and compared to the direct animal movements network.

The results from the bipartite projected networks could clearly demonstrate that the number of edges increased immensely compared to the monopartite animal movements network. This was also the case for the projected feed supply network, although only for 130 farms, which represents 15% of the farms in the network, information about the feed supply was available and thus, most of the farms were isolated in this network representation. This massive increase of edges in the projected network representations can be explained by the fact that contrary to the pyramidal structure of the animal movements network with all movements going downwards in the production chain, the transportation as well as the feed supply network allowed for cyclic movements which enable a re-entry of the movements at the beginning of the production chain via the trucks which transported the animals or the feed between the farms. Also, other studies confirmed the higher number of connections via indirect links compared to the direct animal movements. Bernini et al. [11] stated that although indirect contacts are known to be less infectious than direct contacts [12,36], there are more indirect contacts than direct ones [13,14,15]. Thus, indirect transmission routes can play a potential role in disease transmission [11].

Although the number of edges increased immensely for the two projected network representations in comparison to the animal movements network, the number of network components, the size of the largest connected component, as well as the fragmentation were identical for the animal movements network and the projected transportation network. Here, for the animal movements network and the projected feed supply network without isolates, similar values were obtained. Also, the number of communities was similar. This implies that the network structures were comparable between the three network representations. These resemblances could probably be explained by the spatial distribution of the single nodes. According to Rossi et al. [15], the effect of direct and indirect contacts can occur on different spatial scales. Indirect contacts were responsible for disease transmission on a more local scale, whereas direct contacts could spread pathogens over a longer distance. Also, Salines et al. [19] stated that the swine trade network in France demonstrated geographically clustered communities. They proposed that a geographical compartmentalization could be used to limit the introduction of a disease transmitted via indirect contacts.

Besides all these similarities in the general network parameters, the median centrality parameters revealed clear differences between the three network representations. Moreover, the Spearman rank correlation coefficients between the centrality parameters of the animal movements network and the projected transportation network showed only moderate correlation coefficients and no significant correlation coefficients could be obtained for the projected feed supply network without isolates. This indicates that the ranking of the farms according to the centrality parameters changed between the three network representations. According to Frössling et al. [37] and Dubé et al. [38], for the implementation of targeted control and prevention measurements for disease surveillance programs which are associated with animal movements, node level centrality parameters can help to enhance the informative value and sensitivity of such programs. Thus, knowledge about the network structure can guide the targeted selection of farms in order to limit the disease spread within animal trade networks [39]. However, the centrality parameters can only be used reliably for disease control measures if the impact of different influencing factors, such as the kind of transmission route, the inclusion of additional information as so-called edge weights, or the choice of the observation period is known. Only if the ranking of the farms according to their centrality parameters remained relatively stable, they can be used for the prediction of disease transmission or the implementation of new prevention and control measures. 

Regarding the impact of the interaction between network representation and transmission probability on the number of infected farms, the projected transportation network resulted in the largest epidemic size for which 70% to 97% of the farms became infected. For the projected feed supply network considering the network containing isolated farms, the percentage of infected farms was significantly lower with 13% to 15%. Without isolates, the percentage of infected farms ranged between 84% to 100%. For the animal movements network the lowest number of infected farms were detected with a maximum of 1%. These large differences between the animal movements network and the two projected networks can be explained by the directed nature of the animal movements network with all trade contacts directed downwards the pork supply chain and ending at the abattoirs [3,4,5,17,19]. When these dead-ends are reached, a further disease spread is interrupted. This is the reason that for most studies dealing with the analysis of animal trade networks, abattoirs are excluded from the network due to their dead-end characteristic [4,5,6,17,40,41,42]. In contrast to this, the two projected network representations are undirected allowing also cyclic movements. For the projected transportation network this implies that due to contaminated livestock trucks the disease can be entered again in the production chain. Moreover, Thakur et al. [18] stated that the inclusion of livestock trucks increased the connectivity of farms in the Canadian swine movement network which simultaneously decreased the number of links required to traverse the network from one farm to another. Through indirect contacts via transport companies or feed producers, farms can be connected which are not directly trading animals with each other. In Figure 1, farm A and farm F are not directly trading animals with each other in the animal movements network. However, these two farms are connected with each other in the projected transportation network as they used the same transport company for the transportation of their animals. Furthermore, the study of Salines et al. [19] stated that the potential epidemic size would be larger for an indirectly transmitted disease than for a directly transmitted one. This fact facilitates the spread of diseases within the network, which is confirmed by the results obtained in the present study.

Trucks used for the transportation of animals or feed can be considered as epidemiological units when they are contaminated [17,18]. Unless trucks are not properly cleaned and disinfected, they can potentially spread diseases between farms. Thus, according to Dee et al. [43] appropriate cleaning and disinfection protocols for trucks, as well as biosecurity measures at the farm gate, are essential for limiting the transmission of diseases via indirect connections. However, Lambert et al. [20] stated that in Canadian swine trade networks less than one-third of livestock trucks were cleaned and disinfected between successive transports which facilitates the spread of diseases via indirect connections. Therefore, transport vehicles have to be considered in the analyses, as the potential lack of appropriate cleaning and disinfection facilitates disease transmission, although this inclusion might represent an overestimation of the potential disease transmission and thus a worst-case scenario.

The obtained differences in the epidemiological model based on the three network representations demonstrate clearly the importance of the integration of different transmission routes into epidemiological models as the number of infected farms varied widely between the three network representations. Furthermore, an integration of more than one possible transmission route into the epidemiological models and the network analysis would be beneficial for further analysis. This was not considered in the present study as the focus was laid on the comparison between the three different network representations. As diseases can spread over different transmission routes, the findings of the present study represent a simplification of the spreading process, but allowed a comparison between the results of the network analysis and the simulation model. Moreover, it has to be considered that for all network representations the same epidemiological model was used in order to enhance the comparability of the results. This model assumed the same transmission probability for the different edges, which probably overestimated the true epidemic size for the two projected networks in direct comparison to the animal movements network as already mentioned above. Previous studies showed that for direct animal contacts the transmission probability was highest, followed by the contact with contaminated vehicles and the contact with vehicles transporting products such as feed [8,44,45,46]. According to Dewulf et al. [47], who analyzed the indirect transmission of classical swine fever, livestock trucks contaminated with excretions and secretions of infected animals that are insufficiently cleaned and disinfected, may be an important disease transmission route. Horst et al. [48] stated that based on the classical swine fever epidemic in The Netherlands from 1997 to 1998, contaminated empty livestock trucks were the second most important factor for disease transmission. Although indirect transmission routes usually have a lower transmission probability compared to direct contacts, the huge number of these indirect connections clarify their importance for disease transmission as already discussed above. However, for each disease under investigation, the specific transmission probabilities for the direct, as well as the indirect transmission, have to be chosen appropriately in order to obtain reliable results from the epidemiological models. Although the transmission probability for the projected transportation, as well as the projected feed supply network, is clearly lower compared to the direct animal movements network which was disregarded in the present study, the high number of infected farms is a clear indicator that these transmission routes should be considered for further epidemiological models. 

The present study demonstrated that the inclusion of indirect transmission routes increased immensely the number of infected farms in comparison to the direct animal movements network. As different indirect contacts contribute to a varying content to the extent of epidemics depending on the disease under investigation, further studies should also focus on other indirect transmission routes, such as person contact by e.g., the veterinarian, or shared equipment between the farms. Moreover, it would be beneficial to analyze the different transmission routes as multipartite networks to evaluate the mutual interference and to answer the question of which type of contact is needed or which type of contacts adds significant value to the results of the network analysis as well as the epidemiological models. Furthermore, indirect transmission routes may result in geographically clustered network structures, which could be used for geographical compartmentalization in order to limit disease spread via indirect contacts [19]. 

The present study represents a case study of a pork supply chain of a producer community in Northern Germany. Thus, the results of this study may only be representative of this analyzed animal trade network. However, due to the fact that other pig trade networks focusing on direct animal movements showed similar characteristics (e.g., pyramidal acyclic and directed structure, right-skewed distribution of centrality parameters, heterogeneous contact patterns) [4,5,17,49,50,51], the present results contribute to a great extent to a further understanding of the spreading processes within animal trade networks. Particularly the integration of other transmission routes as performed in the present study into simulation models of the spreading processes within the network based on contact structures is to the authors’ knowledge performed in only a low number of studies for animal trade networks [5,11,13,18,19,20,21,22,23,24,25]. However, the crude assumptions made for the epidemiological model may lead to an underestimation of the final epidemic size.

Another important aspect, which has to be taken into account, is the fact that only information on aggregated trade contacts was analyzed. No information about the temporal development or other transmission routes was included in the model. Also, the frequency of the contacts was not considered in the present study. This illustrates an abstraction of the real trading activities and actual transmission routes in the animal trade network, but it provides an initial insight into the possible disease transmission over different transmission routes. Natale et al. [1] stated that the inclusion of temporal patterns enables taking the chronological order of the trade contacts into account. A disease can only be transmitted from one farm to another if the movements of animals are consequent in time. This can also be applied to the actual rounds of livestock transportation per day so that the livestock trucks visit the farms in a distinctive order [11,19]. If temporal information or information about the actual truck and the rounds which is taken by this truck is known, this information should be included in further analysis as they can picture the actual temporal order of trade contacts in more detail [52]. According to Masuda et al. [53] and Lentz et al. [54], this approach can potentially lead to a more efficient way of analyzing, forecasting, and preventing epidemics. 

## 5. Conclusions

Besides the analysis of the direct animal movements between the farms, the aim of the present study was to integrate further transmission routes via indirect contacts over transport companies and feed producers and to evaluate their impact on the outcome of network analysis and epidemiological models. Although the number of edges increased immensely from the monopartite animal movements network to both projected network representations, the general network parameters showed similar results. In contrast, the centrality parameters revealed significant differences between the three network representations. Moderate correlation coefficients were only obtained between the animal movements network and the projected transportation network. For the results of the epidemiological networks, the number of infected farms was significantly higher for the projected network representations. Whereas in the projected network representations 70% to 100% of the farms became infected in dependence of the underlying transmission probability, in the animal movements network a maximum of 1% of the farms became infected. The results of the present study indicate that the inclusion of indirect transmission routes had an immense impact on the outcome of centrality parameters, as well as on the results of the epidemiological models. Only detailed knowledge of the potential transmission routes can provide a deeper understanding of disease spread via direct and indirect contacts. This knowledge is needed in order to establish control and prevention measurements which are able to inhibit or to interrupt disease spread within animal trade networks.

## Figures and Tables

**Table 1:** Description of the general network parameters and centrality parameters used in the present study.

Parameter	Definition
General network parameters	
Density	The proportion of present edges in comparison to the number of all possible edges [16].
Component	Two nodes are part of the same network component if they are connected by at least one path through the network [27]. A path is thereby defined as a sequence of nodes connected by edges [16].
Fragmentation	The proportion of the number of connected components to the number of nodes in the network [28].
Community	Subset of nodes in which there are significantly more edges as expected by chance (group of preferentially linked nodes) [16,29].
Modularity	Community detection is characterized by the modularity function Q which is maximized (Q_max_) in order to detect the most suitable partition of the network [30].
Centrality parameters	
Degree	Measure of the number of edges directly connected to a node [16].
Betweenness	Extent to which a node lies on the shortest paths between other nodes in the network [10].
Closeness	Mean reciprocal distance from one node to all other reachable nodes in the network [10].

**Table 2:** General network parameters of the animal movements network (AM), the projected transportation network (TR_projected_) and the projected feed supply network (FS_projected_). For the projected feed supply network, the italic numbers in parentheses illustrate the values calculated without isolated farms.

	AM	TR_projected_	FS_projected_
Number of nodes	866	866	866 *(130)*
Number of edges	1884	29,062	3284 *(3284)*
Isolated nodes	0	0	736 *(0)*
Density	0.003	0.075	0.009 *(0.392)*
Fragmentation	0.02	0.02	0.98 *(0)*
Number of components	5	5	737 *(1)*
Size of largest component	856 (98.8%)	856 (98.8%)	130 (15.0%) *(130 (100%))*
Number of communities	18	11	741 *(5)*
Size of largest community	237 (27.4%)	424 (49.0%)	37 (4.3%) *(37 (28.5%))*
Maximum modularity	0.52	0.30	0.24 *(0.24)*

**Table 3:** Median (range) centrality parameters of the animal movements network (AM), the projected transportation network (TR_projected_), and the projected feed supply network (FS_projected_). For the projected feed supply network, the italic numbers in parentheses illustrate the values calculated without isolated farms.

	AM	TR_projected_	FS_projected_
Degree	2 (1 to 204)	35 (1 to 532)	0 (0 to 114) *(63 (6 to 114))*
Betweenness	0 (0 to 0.25)	1.45 × 10^−5^ (0 to 0.20)	0 (0 to 3.28 × 10^−3^) *(0 (0 to 0.15))*
Closeness	0.29 (0.29 to 0.42)	0.44 (0 to 0.68)	0 (0 to 0.13) *(0.65 (0.35 to 0.90))*

**Table 4:** Spearman rank correlation coefficients between the centrality parameters of the animal movement network (AM) and the projected transportation network (TR_projected_) as well as the projected feed supply network (FS_projected_). Values marked with an asterisk (*) are significant (*p* ≤ 0.05). For the projected feed supply network, the italic numbers in parentheses illustrate the values for the network calculated without isolated farms.

	AM-TR_projected_	AM-FS_projected_
N	866	866 *(130)*
Degree	0.61 *	0.41 * *(0.09)*
Betweenness	0.68 *	0.19 * *(0.07)*
Closeness	0.55 *	0.45 * *(0.13)*
